# Genetic determinants of renal scarring in children with febrile UTI

**DOI:** 10.1007/s00467-024-06394-6

**Published:** 2024-05-20

**Authors:** Therese Rosenblad, Magnus Lindén, Ines Ambite, Per Brandström, Sverker Hansson, Gabriela Godaly

**Affiliations:** 1 Section for Pediatric Nephrology, Skåne University Hospital, Lund, Sweden; 2 Department of Pediatrics, Halland Hospital, Halmstad, Sweden; 3 Department of Laboratory Medicine, Lund University, Lund, Sweden; 4 Pediatric Uro-Nephrology Centre, Queen Silvia’s Children’s Hospital, Gothenburg, Sweden; 5 Department of Pediatrics, Institute of Clinical Sciences, Sahlgrenska Academy, University of Gothenburg, Gothenburg, Sweden

**Keywords:** Urinary tract infection, Infants, Renal scarring, Genetic polymorphisms, Long-term consequences, RS risk factor

## Abstract

**Background:**

Febrile urinary tract infections (UTIs) are among the most severe bacterial infections in infants, in which a subset of patients develops complications. Identifying infants at risk of recurrent infections or kidney damage based on clinical signs is challenging. Previous observations suggest that genetic factors influence UTI outcomes and could serve as predictors of disease severity. In this study, we conducted a nationwide survey of infant genotypes to develop a strategy for infection management based on individual genetic risk. Our aims were to identify genetic susceptibility variants for renal scarring (RS) and genetic host factors predisposing to dilating vesicoureteral reflux (VUR) and recurrent UTIs.

**Methods:**

To assess genetic susceptibility, we collected and analyzed DNA from blood using exome genotyping. Disease-associated genetic variants were identified through bioinformatics analysis, including allelic frequency tests and odds ratio calculations. Kidney involvement was defined using dimercaptosuccinic acid (DMSA) scintigraphy.

**Results:**

In this investigation, a cohort comprising 1087 infants presenting with their first episode of febrile UTI was included. Among this cohort, a subset of 137 infants who underwent DMSA scanning was subjected to gene association analysis. Remarkable genetic distinctions were observed between patients with RS and those exhibiting resolved kidney involvement. Notably, the genetic signature indicative of renal scarring prominently featured mitochondrial genes.

**Conclusions:**

In this nationwide study of genetic susceptibility to RS after febrile UTIs in infancy, we identified a profile dominated by mitochondrial polymorphisms. This profile can serve as a predictor of future complications, including RS and recurrent UTIs.

**Graphical abstract:**

**Figure:**
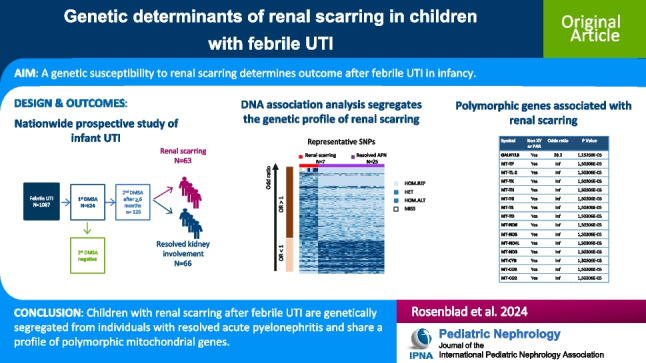
A higher resolution version of the Graphical abstract is available as Supplementary information

**Supplementary information:**

The online version contains supplementary material available at 10.1007/s00467-024-06394-6.

## Introduction

Urinary tract infection (UTI) is a common bacterial infection in childhood, affecting approximately 2% of all infants [1]. Urosepsis and acute pyelonephritis (APN) are the most severe forms of UTI, leading to systemic infections with kidney damage. One potential consequence of APN in childhood is renal scarring (RS). Estimates of the frequency of RS in children vary across populations due to inconsistencies in the timing of acute imaging and follow-up examinations [2]. Nevertheless, population-based studies suggest that 10–26% of children with febrile UTI may suffer from RS [3–6]. The presence of renal involvement can be diagnosed early in some patients post-infection, while in others, scarring is only detected during follow-up, implying that the scarring process can be acute or gradual, possibly influenced by recurrences and other predisposing factors [7–10].

The pathogenesis of RS is subject to debate, as it encompasses parenchymal anomalies, dysplasia (CAKUT), and acquired scarring due to the complex interaction of virulent bacteria and the host's immune response [11, 12]. While vesicoureteral reflux (VUR) increases the risk of RS, scarring can also develop in the absence of VUR [13]. Therefore, VUR must be considered when exploring susceptibility factors for RS. Another well-known risk factor is recurrent UTI and the degree of acute inflammation [10, 14]. Maximum temperature, C-reactive protein (CRP) levels and procalcitonin have been associated with RS, indicating that a severe kidney infection increases the risk of persistent scarring [4, 15, 16]. In conclusion, these observations suggest that various host susceptibility factors, including genetic factors, may predispose a subgroup of children to severe infection and RS [8, 17–20].

Currently, there are no reliable tools for identifying the subset of children with APN who will develop RS. Genes with polymorphic variations, potentially linked to RS, are under investigation [21–23]. However, specific genetic markers indicating susceptibility to scarring have not been conclusively identified. Nevertheless, recent research has associated children with APN as a genetically distinct subgroup of patients with febrile UTI (unpublished observations). Acute febrile UTI is accompanied by increased immune responses in all UTI patients, with a more pronounced activation of innate immunity in the APN group. Additionally, there is an attenuation of adaptive immunity characterized by the inhibition of genes affecting leukocyte development, accompanied by an activation of a lymphocyte exhaustion pathway (unpublished observations). These findings suggest that a specific gene expression profile is associated with APN and kidney involvement as defined by DMSA scans. In this study, DNA analyses were used to determine whether genetic host factors of susceptibility to RS could be identified in infants with febrile UTI. Secondary outcomes aimed to test for genetic host factors predisposing to dilating VUR and recurrent UTI.

## Materials and methods

### Study design

This nationwide multi-center prospective study of infant UTI was conducted at 29 pediatric centers in Sweden, from March 20, 2017, to February 29, 2021. The genetic evaluation of infant UTI was based on a thorough clinical description of the entire course from the first episode of febrile UTI, through the subsequent examinations, and up to one year post-infection. The study was approved by the Swedish Ethical Review Authority (Dnr 2015/884, Dnr 2015/884, and Dnr 2016/799). Written and oral information was provided to the children’s caregivers, and participation required written informed consent.

### Patients

Infants under one year of age with a first episode of febrile UTI were eligible for inclusion. Exclusion criteria included overt urogenital malformations or ongoing catheter therapy. Children were enrolled from emergency units when antibiotic treatment was initiated due to a clinical diagnosis of suspected UTI. All participants received treatment according to local practice, in line with the Swedish guidelines for managing febrile UTI in children under 2 years of age [24]. Diagnostic criteria for febrile UTI were a temperature exceeding ≥ 38.0°C and a positive urine culture indicating the presence of a single uropathogenic bacterium (any growth in urine obtained through suprapubic aspiration, ≥ 10^4^ cfu/mL in catheterized urine, or ≥ 10^5^ cfu/mL in mid-stream clean-catch urine). DNA samples were collected upon inclusion. Data regarding clinical and laboratory findings were recorded at each of the 29 collaborating centers and entered into a database at the University of Gothenburg at two time points: after the initial evaluation and one year following the infection. The results of follow-up examinations, prophylactic antibiotic treatment, surgical interventions, and new episodes of febrile UTI were reported.

### Imaging

Infants diagnosed with febrile UTI underwent ultrasound examinations (*n* = 1081) to identify urinary tract dilatation and rule out significant malformations. DMSA scintigrams were conducted to diagnose kidney involvement and were analyzed locally in a selected group of children with one or more risk factors, such as CRP ≥ 70 mg/L, creatinine > 30 μmol/l, or non-*E. coli* growth in urine culture. Decreased kidney uptake in examinations conducted after the acute infection, regardless of the time interval from the infection, was considered as kidney involvement (first DMSA positive). Acute pyelonephritis (APN) was defined as decreased kidney uptake in examinations performed within seven days from diagnosis. In children with kidney involvement on the first DMSA scan, RS was diagnosed through a second DMSA scan conducted ≥ 6 months after the infection, based on persistent kidney involvement or reduced differential function < 45%. If the first DMSA scan was normal, and the infants did not experience any new episodes of febrile UTI, they were considered to have normal kidneys during the follow-up period [3]. Voiding cystourethrography (VCUG) was performed in selected patients, primarily in those with dilatation on kidney ultrasound or reduced differential function on DMSA. Vesicourethral reflux (VUR) was graded 1–5 following the recommendations of the International Reflux Study in Children [25]. The maximum VUR grade was reported for children who underwent more than one VCUG examination and the highest VUR grade for those with bilateral VUR. VUR grades 0–2 are defined as no VUR or non-dilating VUR, while VUR grades 3–5 are defined as dilating VUR. Individuals diagnosed with kidney involvement at the first DMSA scan, who also had follow-up examination, were included in calculations of RS in comparison to VUR grade and UTI recurrences.

### Laboratory parameters and exome genotyping

CRP, creatinine, urine dipstick, and urine culture were included in the initial evaluation, as stipulated in the Swedish national guidelines. Blood samples were collected for a genetic evaluation of RS in children with UTI. DNA was extracted from heparinized peripheral blood using the QIAamp DNA Blood mini kit, and samples were analyzed via exome genotyping using Illumina Infinium Exome bead chip technology.

### Statistical and bioinformatics analysis

SPSS software version 27 was used to analyze the clinical data. Patient criteria were analyzed by Fisher’s exact test for group comparisons with two dichotomous variables, the *χ*^2^ test for ordered categorical variables and for large sample sizes an independent *t* test for continuous variables and a non-parametric Mann-Whitney’s test for skewed or smaller sample sizes. The post hoc Kruskal-Wallis test was used to compare more than two independent groups regarding continuous or ordered variables. *p* < 0.05 was considered significant. Significant values (*p* values of overlap) were calculated by Fisher’s exact test, and *p* values were adjusted for multiple testing.

The primary outcome was the genetic association with RS following acute febrile UTI. DNA association analysis was performed using allelic frequency tests and odds ratio (OR) calculations to compare patient groups based on the second DMSA outcome: (1) RS compared to resolved APN and (2) RS compared to first DMSA negative (i.e., no APN). For the secondary outcomes, we also compared (3) VUR grade 0–2 compared to VUR grade 3–5 and (4) recurrent UTI compared to no recurrences for the DNA association analysis.

For all association analyses, OR values and *p* values were calculated for each single nucleotide polymorphism (SNP) using Fisher’s exact test using the *fisher.test* function in R v.4.1.3 [26]. Variants with *p* values < 0.01 were considered significant. To summarize the association analysis results on the gene level for each gene, the SNP with the lowest *p* value was selected as the representative SNP (< 0.005). The genes with representative SNPs were selected to be visualized with heatmaps. The heat maps were generated in R using the *pheatmap* v.1.0.12 R-package [27]. The sample ordering for the heat maps was done based on DMSA status and columns on mean log2 ORs for DMSA-positive group. Furthermore, a principal component analysis (PCA) plot, using the *prcomp* function in R, was used to show clusters of samples based on their similarity. Variants located in pseudoautosomal regions (PARs) of the XY chromosome were included, while non-PARs were omitted to avoid gender biases in the analysis due to hemizygous male genotypes. SNPs were annotated with chromosomal location, gene name, reference allele/alternative allele, and gene type using Variant Effect Predictor (VEP) [28]. To estimate the prevalence of the SNPs in the Swedish population, the allele frequencies were retrieved from the SweGen database [29].

Pathway analysis was performed using IPA (QIAGEN Inc., https://www.qiagenbioinformatics.com/products/ingenuity-pathway-analysis) of SNPs and included differences in gene associations with *p* values (*p* < 0.005). Genes from the IPA Knowledge database were used as a background reference, and the results analyzed by IPA were presented in terms of canonical pathways, major regulators, upstream regulators, disease, and toxicity functions.

## Results

### Patient characteristics

**Fig.1:**
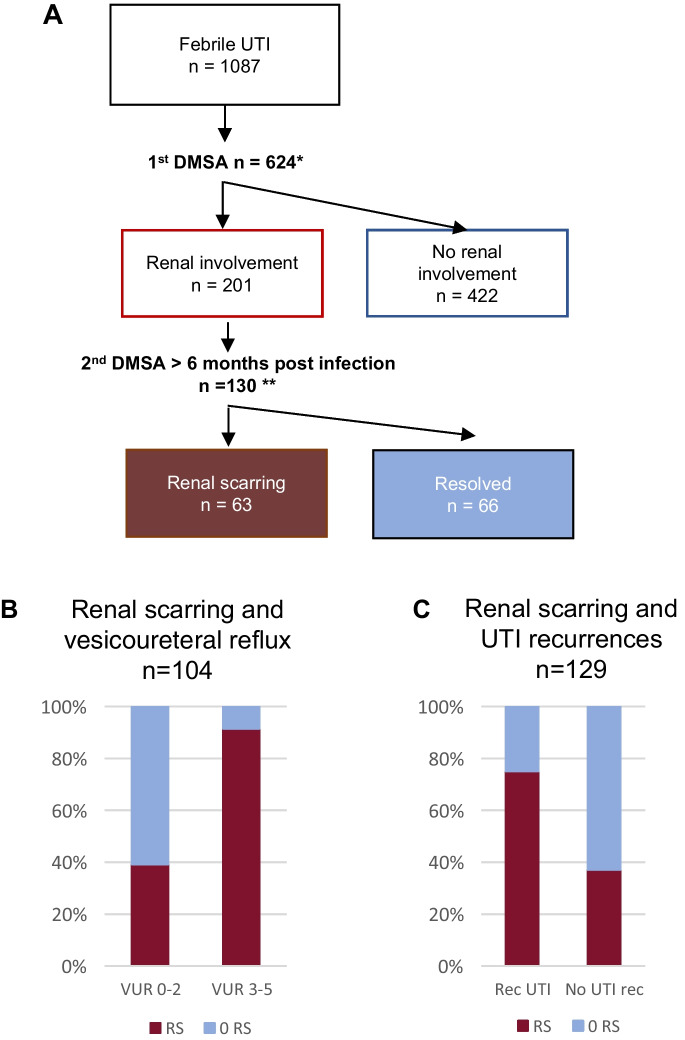
**A** Flow chart depicting the inclusion criteria and outcomes of DMSA scans, identifying infants with kidney involvement after the first DMSA and renal scarring (RS) after the second DMSA evaluations (one result is missing* and one result is excluded due to nephrectomy**). **B** Bar chart illustrating a higher incidence of RS in infants diagnosed with VUR grade 3–5 compared to those with VUR grades 0–2 (*p* < 0.001). **C** Bar chart indicating that RS is more prevalent among children who experienced UTI recurrences (*p* < 0.001)

Among the 1087 infants diagnosed with febrile UTI, an initial DMSA scan was administered to 624 infants (Figure 1A), and subsequently, a DNA association analysis was carried out on a subset of 137 infants. Table 1 displays the age, gender distribution, maximum temperature, and maximum CRP levels for infants not subjected to DMSA scan (group I), infants with a first DMSA examination but not included in genetic evaluation (group II), and infants examined by a first DMSA and included in genetic evaluation (group III). Infants included in DNA evaluation were representative for children subjected to a first DMSA scan.

**Table 1:** The clinical characteristics of the core population of infants diagnosed with febrile urinary tract infection (UTI) were studied, with a total of 1087 subjects (*N*=1087). Three groups were evaluated based on the selection for DMSA examination and DNA analysis

	Group I Infants with febrile UTI, not investigated by DMSA (*n*=462)	Group II Infants with febrile UTI investigated with 1^st^ DMSA, but no DNA analysis performed (*n*=488)*	Group III Infants with febrile UTI investigated with 1^st^ DMSA and subjected to DNA analysis (*n*=137)*
Gender distribution boys/girls	216/246	205/283	65/72
Age at infection, months (mean)	4.0	4.4	4.1
Maximum CRP, mg/L (mean)	50.3	105.0	113.5
Maximum temperature, ℃ (mean)	39.2	39.5	39.5

^*^Group comparisons showed no differences in age, maximum CRP levels, or maximum temperature between groups II and III (*p* = non significant)

### Kidney involvement in first DMSA and renal scarring at follow-up DMSA scan

A total of 201 out of 623 patients (32.3%) showed signs of kidney involvement. Among those who underwent the first DMSA scan within seven days, 57 out of 90 (63.3%) were diagnosed with kidney involvement and classified as having APN. Furthermore, RS was identified in 63 out of 129 (43.2%) patients who underwent a second DMSA scan ≥ 6 months from infection (see Figure 1A).

### Gene association in relation to renal scarring

**Fig. 2:**
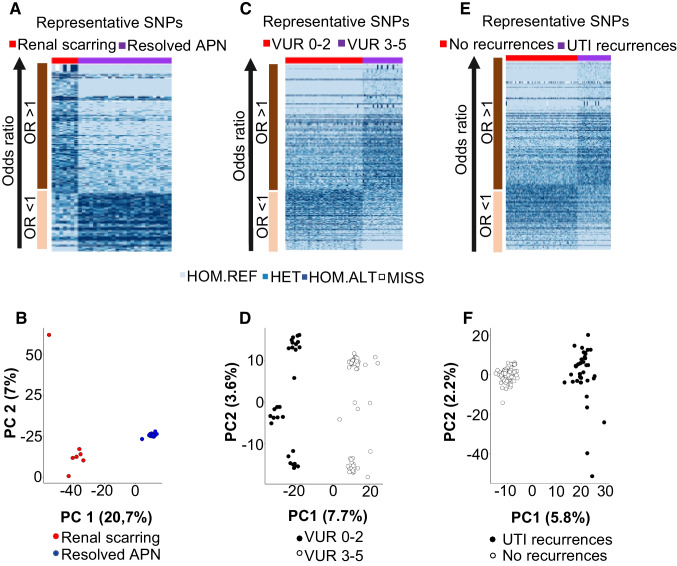
**A** Heatmap illustrating the segregation of SNPs based on an allelic frequency test (*p* < 0.005) and calculation of odds ratio (OR), comparing infants with renal scarring (RS) after acute pyelonephritis (APN) (*n* = 7) and infants with resolved APN (*n* = 25). **B** Principal component analysis plot depicting the genetic differences between the two groups with RS and resolved APN. **C, D** Similar analyses were conducted for the groups with vesicoureteral reflux (VUR) grades 0–2 (*n* = 54), VUR grades 3–5 (*n* = 28), and **E, F** infants with recurrent febrile UTI (*n* = 36) and infants without recurrences (*n* = 77)

Out of 90 children who underwent their first DMSA scan within 7 days of infection, exome genotyping was performed on 56 patients (32 with positive and 24 with negative DMSA results) (Supplementary Figure 1A). We compared significant SNPs (with a cutoff of *p* < 0.005) and calculated individual OR values for each variant in the population, then sorted them into heat maps. The first analysis focused on SNPs associated with RS (*n* = 7) in comparison to those associated with resolved APN (*n* = 25) (Figure 2A). This analysis revealed a distinct separation of variants, with alternative alleles being more prevalent in the scarring group. Group distinctions were further demonstrated in a PCA plot (Figure 2B). In a second analysis of significant SNPs associated with RS (*n* = 7) and the no-APN group (*n* = 24) (with the same *p* < 0.005 cutoff), we also observed a clear genetic differentiation between the two groups (Supplementary Figure 1B).

### Association of mitochondrial genes found in renal scarring

Gene association analysis identified 582 SNPs significantly more common in RS, with the 25 most significant ones listed in Table 2. Among these, 22 SNPs are linked to mitochondrial function, including *ND-4-6*, *ND4L*, *COX I-III*, *ATPase6*, and *ATPase8*, all playing roles in mitochondrial respiratory complexes.

**Table 2:** Genes associated with renal scarring (RS). Polymorphic genes identified through DNA association analysis (allelic frequency test *p* < 0.005) show a prevalence of mitochondrial genes among the most significant ones linked to RS in both analyses, when compared to resolved APN

Symbol	Non-XY or PAR	Odds ratio	*p* value
GALNT18	Yes	28.2	1.23268*E* − 05
MT-TP	Yes	Inf	1.50306*E* − 05
MT-TL-2	Yes	Inf	1.50306*E* − 05
MT-TK	Yes	Inf	1.50306*E* − 05
MT-TH	Yes	Inf	1.50306*E* − 05
MT-TG	Yes	inf	1.50306*E* − 05
MT-TE	Yes	Inf	1.50306*E* − 05
MT-TD	Yes	Inf	1.50306*E* − 05
MT-ND6	Yes	Inf	1.50306*E* − 05
MT-ND5	Yes	Inf	1.50306*E* − 05
MT-ND4L	Yes	Inf	1.50306*E* − 05
MT-ND3	Yes	Inf	1.50306*E* − 05
MT-CYB	Yes	Inf	1.50306*E* − 05
MT-CO3	Yes	Inf	1.50306*E* − 05
MT-CO2	Yes	Inf	1.50306*E* − 05
MT-CO1	Yes	Inf	1.50306*E* − 05
MT-ATP8	Yes	Inf	1.50306*E* − 05
MT-ATP6	Yes	Inf	1.50306*E* − 05
MT-TR	Yes	Inf	1.50306*E* − 05
MT-TS1	Yes	Inf	1.50306*E* − 05
MT-ND4	Yes	Inf	1.50306*E* − 05
MT-TS2	Yes	Inf	1.50306*E* − 05
MT-TT	Yes	Inf	1.50306*E* − 05
RP11-600K15.1	Yes	18.33333333	3.41825*E* − 05
THSD4	Yes	22	3.91023*E* − 05

*Infinitive

These significant SNPs from DNA association analysis comparing children with RS to children with resolved APN were further analyzed using IPA (Table 3). Canonical pathway analysis revealed ten pathways associated with RS (cutoff *p* < 0.005 and adjusted *p* value < 0.05). IPA comparing RS to resolved APN highlighted those genes causing mitochondrial dysfunction and oxidative phosphorylation showed the strongest association with scarring (cutoff *p* < 0.005 and adjusted *p* value < 0.05). This was followed by chondroitin sulfate biosynthesis, dermatan sulfate synthesis, and FGF signaling pathways (Supplementary Table 1). Among the most significant upstream regulators, IPA of RS compared to resolved APN groups (*p* = 0.005, adjusted *p* value < 0.05) confirmed the dominance of mitochondrial genes (Supplementary Table 2).

**Table 3:** Genes associated with renal scarring (RS) were compared between cases of RS and those without acute pyelonephritis (no APN) indicated by a negative first DMSA examination. Mitochondrial genes dominate among the most significant genes associated with RS in both analyses

Symbol	Non-XY or PAR	Odds ratio	*p* value
SH3BP4	Yes	62.66666667	7.20407*E* − 06
MT-ATP8	Yes	Inf*	1.84564*E* − 05
MT-TT	Yes	Inf	1.84564*E* − 05
MT-TS2	Yes	Inf	1.84564*E* − 05
MT-TS1	Yes	Inf	1.84564*E* − 05
MT-TR	Yes	inf	1.84564*E* − 05
MT-TP	Yes	Inf	1.84564*E* − 05
MT-TL2	Yes	Inf	1.84564*E* − 05
MT-TK	Yes	Inf	1.84564*E* − 05
MT-ATP6	Yes	Inf	1.84564*E* − 05
MT-TE	Yes	Inf	1.84564*E* − 05
MT-TH	Yes	Inf	1.84564*E* − 05
MT-ND6	Yes	Inf	1.84564*E* − 05
MT-ND5	Yes	Inf	1.84564*E* − 05
MT-ND4L	Yes	Inf	1.84564*E* − 05
MT-ND4	Yes	Inf	1.84564*E* − 05
MT-ND3	Yes	Inf	1.84564*E* − 05
MT-CYB	Yes	Inf	1.84564*E* − 05
MT-CO3	Yes	Inf	1.84564*E* − 05
MT-CO2	Yes	Inf	1.84564*E* − 05
MT-CO1	Yes	Inf	1.84564*E* − 05
MT-TD	Yes	Inf	1.84564*E* − 05
MT-TG	Yes	Inf	1.84564*E* − 05
RP11-475O6.1	Yes	0.054545455	3.36819*E* − 05
RP11-444D3.1	Yes	0.034965035	4.5158*E* − 05

^*^Infinitive

Further examination of these mitochondrial genes revealed a predominant association with various forms of cancer rather than inflammatory conditions (Supplementary Table 3).

### Involvement of mitochondrial genes distinguishes infants with renal scarring

To investigate whether infants with RS can be genetically distinguished from children with a first negative DMSA, we conducted another DNA comparison. We identified 524 polymorphic genes associated with RS (*p* < 0.005). The strongest association was found in the ubiquitously expressed *SH3BP4*, which is considered to function as a transferrin receptor involved in cell growth regulation, proliferation, and autophagy [30]. Additionally, 22 polymorphic SNPs were of mitochondrial origin, similar to the genes found in the RS analysis above (see Table 2B). Using IPA, we identified 12 canonical pathways associated with RS (*p* < 0.005 adjusted to *p* < 0.05), with the most significant pathways related to mitochondrial dysfunction and oxidative phosphorylation (see Supplementary Table 4).

The dominance of mitochondrial involvement was further confirmed by a combined analysis of common polymorphic genes among infants with RS compared to infants with resolved APN and infants with a first negative DMSA. When comparing the 150 most significant SNPs (*p* < 0.005) in these two groups, we found that 22 out of 50 shared genes were involved in mitochondrial function (see Table 4).

**Table 4:** Fifty common polymorphic genes associated with RS (extracted from two gene association analysis: RS in comparison to resolved APN and RS in comparison to no APN (*p* < 0.005))

Gene	Official name	Function
MT-TP	Mitochondrially encoded tRNA proline	tRNA*
MT-TL2	Mitochondrially encoded tRNA leucine 2	tRNA
MT-TK	Mitochondrially encoded tRNA lysine	tRNA
MT-TH	Mitochondrially encoded tRNA histidine	tRNA
MT-TG	Mitochondrially encoded tRNA glycine	tRNA
MT-TE	Mitochondrially encoded tRNA glutamic acid	tRNA
MT-TD	Mitochondrially encoded tRNA aspartic acid	tRNA
MT-ND6	Mitochondrially encoded NADH dehydrogenase 6	tRNA
MT-ND5	Mitochondrially encoded NADH dehydrogenase 5	tRNA
MT-ND4L	Mitochondrially encoded NADH 4L dehydrogenase	tRNA
MT-ND3	Mitochondrially encoded NADH dehydrogenase 3	tRNA
MT-CYB	Mitochondrially encoded cytochrome b	tRNA
MT-CO3	Mitochondrially encoded cytochrome c oxidase III	tRNA
MT-CO2	Mitochondrially encoded cytochrome c oxidase II	tRNA
MT-CO1	Mitochondrially encoded cytochrome c oxidase I	tRNA
MT-ATP8	Mitochondrially encoded ATP synthase 8	tRNA
MT-ATP6	Mitochondrially encoded ATP synthase 6	tRNA
MT-TR	Mitochondrially encoded tRNA arginine	tRNA
MT-TS1	Mitochondrially encoded tRNA serine 1	tRNA
MT-ND4	Mitochondrially encoded NADH dehydrogenase 4	tRNA
MT-TS2	Mitochondrially encoded tRNA serine 2	tRNA
MT-TT	Mitochondrially encoded tRNA threonine	tRNA
SEC11C	SEC11 homolog C, signal peptidase complex subunit	Protein coding
TWIST2	Twist family bHLH transcription factor 2	Protein coding
FGF12	Fibroblast growth factor 12	Protein coding
RP11-475O6.1	Unknown	lncRNA**
RP11-650J17.1	Non-coding gene	ncRNA***
THSD4	Thrombospondin type 1 domain containing 4	Protein coding
AC007682.1	Probable NRXN1 divergent transcript	
NRXN1	Neurexin 1	Protein coding
ZBTB20	Zinc finger and BTB domain containing 20	Protein coding
CSMD1	CUB and Sushi multiple domains 1	Protein coding
LINC00320	Long intergenic non-protein coding RNA 320	ncRNA
KB-1930G5.4	Unknown	
NT5C2	5′-nucleotidase, cytosolic II	Protein coding
MARCKSL1P1	MARCKS-like 1 pseudogene 1	Pseudo
KIRREL3	Kirre-like nephrin family adhesion molecule 3	Protein coding
CGNL1	Cingulin like 1	Protein coding
B3GAT1	Beta-1,3-glucuronyltransferase 1	Protein coding
GLB1L2	Galactosidase beta 1 like 2	Protein coding
TRIO	Trio Rho guanine nucleotide exchange factor	Protein coding
AGBL1	AGBL carboxypeptidase 1	Protein coding
DPYSL2	Dihydropyrimidinase like 2	Protein coding
CASC15	Cancer susceptibility 15	ncRNA
IMPA2	Inositol monophosphatase 2	Protein coding
C6orf132	Chromosome 6 open reading frame 132	Protein coding
GJA4	Gap junction protein alpha 4	Protein coding
SMIM12	Small integral membrane protein 12	Protein coding
RP11-650J17.2	Unknown	lncRNA
ANK2	Ankyrin 2	Protein coding

^*^Transfer RNA

^**^Long non-coding RNA

^***^Non-coding RNA

### Vesicoureteral reflux

VCUG was performed at least once post-infection in 236 children who also were investigated by a DMSA scan. Among these, 137/236 (58.1%) infants showed no VUR, while 23/236 (9.7%) were diagnosed with non-dilating VUR (VUR grades 1–2), and 76/236 (32.2%) had dilating VUR (VUR grade 3-5). In the group of patients with kidney involvement who underwent a follow-up DMSA scan (*n* = 129), 104 were also investigated with VCUG. RS was more common in children with VUR grades 3, 4, and 5 (100%, 90.0%, 87.5%, respectively) than in those with VUR grades 1 and 2 (0% and 71.4%) (Figure 1B). RS was also diagnosed in children without VUR (37.3%) (*p* < 0.001).

### Gene association in relation to VUR

In this analysis, we compared 54 infants with VUR grades 0–2 to 28 infants with VUR grades 3–5. Infants included in the DNA evaluation were randomly selected and demonstrated a similar distribution of dilating VUR (34.1%) as described above in children subjected to both VCUG and DMSA. As in the RS analysis, we observed a clear distinction between the groups of VUR grades 0–2 and VUR grades 3–5. This separation is visualized in the heatmap and PCA plots (Figure 2C, D).

DNA analysis identified 893 polymorphic SNPs associated with dilating VUR (*p* < 0.005). A mitochondrial profile was evident in 22 of the 50 most significant genes. Polymorphic genes previously linked to CAKUT, such as *GRIP1*, were also associated with children with dilating VUR [31]. The top 50 genes associated with dilating VUR are listed in Supplementary Table 5. IPA canonical pathway analysis identified mitochondrial dysfunction (Supplementary Table 6), and the upstream regulators were dominated by mitochondrial genes (Supplementary Table 7).

### Recurrent UTI

In our study, 136 out of 622 infants investigated by a first DMSA (21.9%) experienced recurrent febrile UTIs. Among the 129 children who underwent a second DMSA, RS was more common in the group with recurrent UTIs compared to children without UTI recurrences (75.0% compared to 37.1%, Figure 1C, *p* < 0.001).

### Gene association in relation to recurrent febrile UTI

In our DNA association analysis comparing infants with reported recurrences of febrile UTI (*n* = 36) to infants without UTI recurrences (*n* = 77), we identified 814 polymorphic SNPs. The top 25 genes associated with UTI recurrences are presented in Supplementary Table 8. A trend of group separation was observed, although it was less distinct than the clear separation observed in the previous RS compared to resolved APN analysis (Figure 2 E-F). This group showed heterogeneity with no dominant immunity genes. The most related genes were *Peli2*, linked to IL-1 family signaling, and the transcription factor *ZNF195*, which has a kidney association (www.genecards.org). When the allele counts of the representative SNPs for these 25 genes were compared against the SweGen database, it was found that many of them exhibited significantly different allele proportions also on the populations level.

IPA of the canonical pathways identified 46 pathways related to recurrent UTI, including genes associated with mitochondrial dysfunction (Supplementary Table 9). IPA-Tox analysis revealed gene expression changes associated with UTI recurrence genes, mainly related to liver and heart diseases, but also nephrosis (Supplementary Table 10) [32]. Network analysis indicated an association with connective tissue disorders and organismal abnormalities among the ten most significant diseases and functions annotated by IPA.

### Common risk genes of RS, dilating VUR and recurrent UTI

**Fig. 3:**
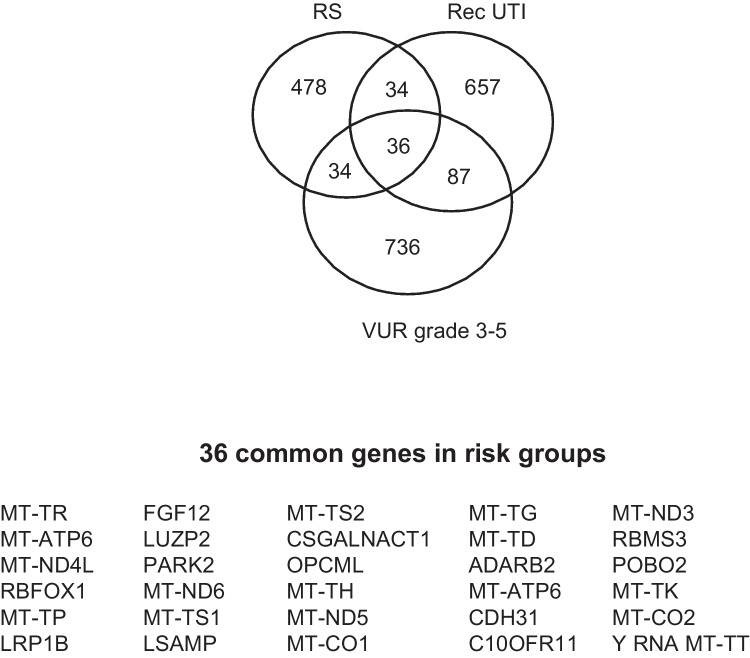
Venn diagram and corresponding table showing 36 common risk genes of renal scarring (RS), recurrent urinary tract infection (UTI), and dilating vesicoureteral reflux (VUR), involving mitochondrial genes and FGF12 (*p* < 0.005)

To identify possible shared risk genes, a Venn analysis was performed on the significant genes associated with RS, dilating VUR, and recurrent UTI. We identified 36 common genes (*p* < 0.005) considered as RS susceptibility genes, of which 23 are mitochondrial (Figure 3). IPA of all the common genes of RS, VUR grades 3–5, and recurrent UTI was also conducted. Affected canonical pathways are presented in Supplementary Table 11 and include mitochondrial dysfunction. Annotation of toxic functions describes major organ involvement, with cardiac diseases being most dominant, followed by liver and mitochondrial diseases, in addition to renal fibrosis and glomerular disease (Supplementary Table 12).

## Discussion

In our population, RS was linked with a mitochondrial gene profile, distinguishing it from the profile of children with resolved APN and those without any kidney involvement in the first DMSA. The association of polymorphic mitochondrial genes with RS in children has not been previously demonstrated, but the clinical relevance is supported by research describing the role of mitochondria in acute kidney injury and in chronic kidney diseases such as focal segmental glomerulosclerosis and tubulopathy [33–35]. Since mitochondrial genes affect most organs, they play a role in the heterogeneity of disease phenotypes in children [36]. A recent publication describes a threshold level of mutated mitochondrial DNA, which varies between different tissues depending on energy demand. This makes the brain, heart, and kidneys especially sensitive [35], which could possibly explain why many annotations by IPA link diseases and toxic functions to these organs. Additionally, in our study, canonical pathway analysis further supports the association of mitochondrial engagement with RS. The pathogenesis of scarring could involve sensitivity to infection due to affected mitochondrial function. The mitochondrial role in immunity encompasses the production of reactive oxygen species (ROS) and other signaling molecules such as mitochondrial constituents and metabolic products in the progression of inflammatory disorders [37–39]. For example, the leakage of mtDNA is known to increase the levels of ROS, leading to hyperactivation of inflammatory responses resulting in kidney damage and pathology [33]. This is intriguing given previous studies showing that a distinctive genetic APN profile is related to hyperinflammation in combination with inhibited lymphocyte maturation (unpublished observations). In our population, we also found *XRCC* associated with RS. This gene is important for DNA repair and has previously been linked to kidney failure risk [37–39], thus connecting RS susceptibility to kidney failure.

In this study, we focused on identifying genetic traits associated with susceptibility to RS following febrile UTI. We observed a clear genetic distinction between patients with RS and those with resolved APN. We specifically chose to include only children who had undergone two DMSA scans. Furthermore, since participants were managed according to the local routines of each center with variable access to acute DMSA scan, the number of DNA samples from patients with a first DMSA examination within seven days from UTI diagnosis was relatively low. However, we still discovered a highly significant group of genes associated with RS, which strengthens our results.

We observed associations between dilating VUR, recurrent UTI, mitochondrial polymorphisms, and dysfunction. These findings bridge the genetic results from RS to the clinical risk groups [5, 10, 14, 40–43]. Within our cohort, 32.2% of children examined by VCUG showed dilating VUR, and 137 out of 623 (22.0%) experienced recurrent UTI. As anticipated, RS was more prevalent among infants with dilating VUR and recurrent UTI, prompting us to conduct a secondary gene association analysis. Comparing infants with dilating VUR to those without shed light on the mitochondrial role, particularly the upstream regulators, all of which were implicated in mitochondrial functions. This was also reflected in the affected canonical pathways. When we examined the DNA for disease and toxic functions using IPA, the affected pathways were related to other energy-sensitive organs such as liver and heart diseases. It's worth noting that several polymorphic genes associated with dilating VUR in our population were previously identified in CAKUT [44]. While RS is linked with VUR, it can also occur in the absence of VUR. This supports the notion that both impairment of kidney development and infection-induced responses play crucial roles in RS pathogenesis. Common genes among RS, dilating VUR, and recurrent UTI demonstrated a clear mitochondrial dominance. Additionally, *FGF12*, a member of the FGF family linked to renal fibrosis and various cellular processes, was identified [45–47]. Serum b-FGF levels have been proposed as a VUR marker in RS but not yet implemented [48].

The choice of significance level significantly influences bioinformatic findings. Using a lower threshold (*p* < 0.05), genes previously linked to kidney disease (*TINAG* and *COL4A1*) were found among common risk genes [49–51]. A literature comparison of CAKUT-related genes identified five common genes reported in all three groups—RS, VUR grades 3–5, and recurrent UTI [52]. Furthermore, *PAX2* was identified as a shared gene between RS and dilating VUR. Mutations in the *PAX2* gene have previously been implicated in kidney abnormalities, including VUR [53–55]. The hepatocyte nuclear factor 1β (HNF1β), associated with kidney pathology in CAKUT patients, was detected in the RS group [56–58]. Finally, vascular endothelial growth factors (*VEGF A* and *C*) were significant in RS (*p* < 0.05). *VEGF* gene variants have previously been suggested as potential genetic markers for VUR and UTI [59, 60].

The interpretation of our findings presents a considerable challenge. Our primary objective was to identify potential genetic determinants in children predisposed to RS. Clinically, there is an overlap between scarring due to congenital malformations and infection-induced RS, but in our material, we are not able to separate these conditions with certainty. We believe that these infants are representative of the group of children where kidney defects are detected after febrile UTI and hypothesize that concurrent factors like acute inflammation and CAKUT may contribute to the scarring susceptibility after febrile UTI. In our investigation, we have unveiled a cluster of genes associated with crucial biological processes, including metabolism, mitochondrial function, cell differentiation, and developmental pathways. Notably, the pronounced involvement of mitochondrial function stands out in our results. While additional research involving the genetic underpinnings of RS remains imperative, our current findings posit that mitochondrial function plays a pivotal role in rendering individuals more susceptible to RS when carrying specific mitochondrial function-affecting polymorphisms. It is noteworthy to mention that bilateral RS leads to enduring complications such as hypertension, a reduced GFR, and an increased risk of progressing to kidney failure [61–64]. In a follow-up study of patients after childhood UTI, Jacobson et al. found that even unilateral RS is associated with increased renin activity levels, diastolic hypertension and decreased GFR compared to controls, but small unilateral scars are not associated with long-term complications [65, 66]. Our findings substantially advance our understanding of the fundamental mechanisms contributing to the pathogenesis of RS.

The absence of reliable RS predictive markers can lead to unwarranted and potentially harmful diagnostic procedures in infants followed up after febrile UTI, increasing their exposure to ionizing radiation and incurring avoidable costs. Hence, the identification of susceptible individuals is of paramount importance to facilitate personalized and effective management strategies in the future. The development of novel tools for the early detection of RS susceptibility is imperative. As suggested by Khan et al. for CKD, the consideration of implementing a polygenic risk score is a prospective avenue for identifying children at risk of experiencing long-term complications following a febrile UTI [67].

### Supplementary information

Below is the link to the electronic supplementary material.

Graphical abstract (PPTX 175 KB)

Supplementary file2 (PPTX 129 KB)

Supplementary file3 (DOCX 57 KB)

## Data Availability

The gene association analysis is available from the lead author upon request.
