# Supplementary material for: Genetic determinants of renal scarring in children with febrile UTI
Source: Pediatr Nephrol. 2024 May 20;39(9):2703–15. doi: 10.1007/s00467-024-06394-6 (PMC11272715; doi:10.1007/s00467-024-06394-6)
Supplement: Supplementary file 1 — Graphical abstract (PPTX 175 KB) [file 467_2024_6394_MOESM1_ESM.pptx]

## Slide 1
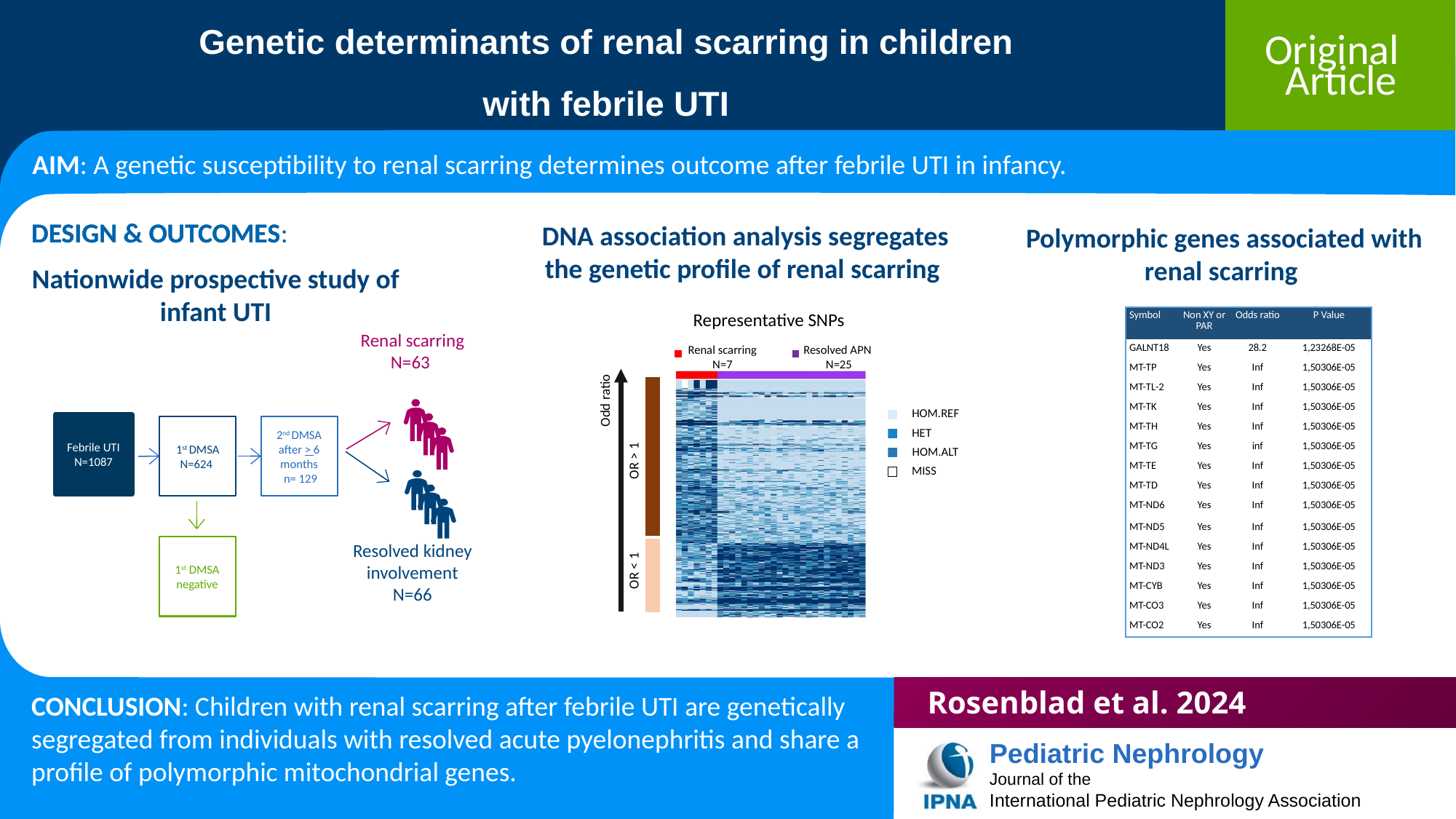

Genetic determinants of renal scarring in children
with febrile UTI
AIM: A genetic susceptibility to renal scarring determines outcome after febrile UTI in infancy.
DESIGN & OUTCOMES:
DESIGN & OUTCOMES
DNA association analysis segregates the genetic profile of renal scarring
Polymorphic genes associated with renal scarring
Nationwide prospective study of infant UTI
Representative SNPs
Renal scarring
N=7
Resolved APN
N=25
Odd ratio
OR > 1
OR < 1
HOM.REF
HET
HOM.ALT
MISS
| Symbol | Non XY or PAR | Odds ratio | P Value |
| --- | --- | --- | --- |
| GALNT18 | Yes | 28.2 | 1,23268E-05 |
| MT-TP | Yes | Inf | 1,50306E-05 |
| MT-TL-2 | Yes | Inf | 1,50306E-05 |
| MT-TK | Yes | Inf | 1,50306E-05 |
| MT-TH | Yes | Inf | 1,50306E-05 |
| MT-TG | Yes | inf | 1,50306E-05 |
| MT-TE | Yes | Inf | 1,50306E-05 |
| MT-TD | Yes | Inf | 1,50306E-05 |
| MT-ND6 | Yes | Inf | 1,50306E-05 |
| MT-ND5 | Yes | Inf | 1,50306E-05 |
| MT-ND4L | Yes | Inf | 1,50306E-05 |
| MT-ND3 | Yes | Inf | 1,50306E-05 |
| MT-CYB | Yes | Inf | 1,50306E-05 |
| MT-CO3 | Yes | Inf | 1,50306E-05 |
| MT-CO2 | Yes | Inf | 1,50306E-05 |
Renal scarring
N=63
Resolved kidney involvement
N=66
Febrile UTI
N=1087
1st DMSA
N=624
2nd DMSA after > 6 months
 n= 129
1st DMSA negative
Rosenblad et al. 2024
CONCLUSION: Children with renal scarring after febrile UTI are genetically segregated from individuals with resolved acute pyelonephritis and share a profile of polymorphic mitochondrial genes.
