# Supplementary material for: Genetic determinants of renal scarring in children with febrile UTI
Source: Pediatr Nephrol. 2024 May 20;39(9):2703–15. doi: 10.1007/s00467-024-06394-6 (PMC11272715; doi:10.1007/s00467-024-06394-6)
Supplement: Supplementary file 2 — Supplementary file2 (PPTX 129 KB) [file 467_2024_6394_MOESM2_ESM.pptx]

## Slide 1
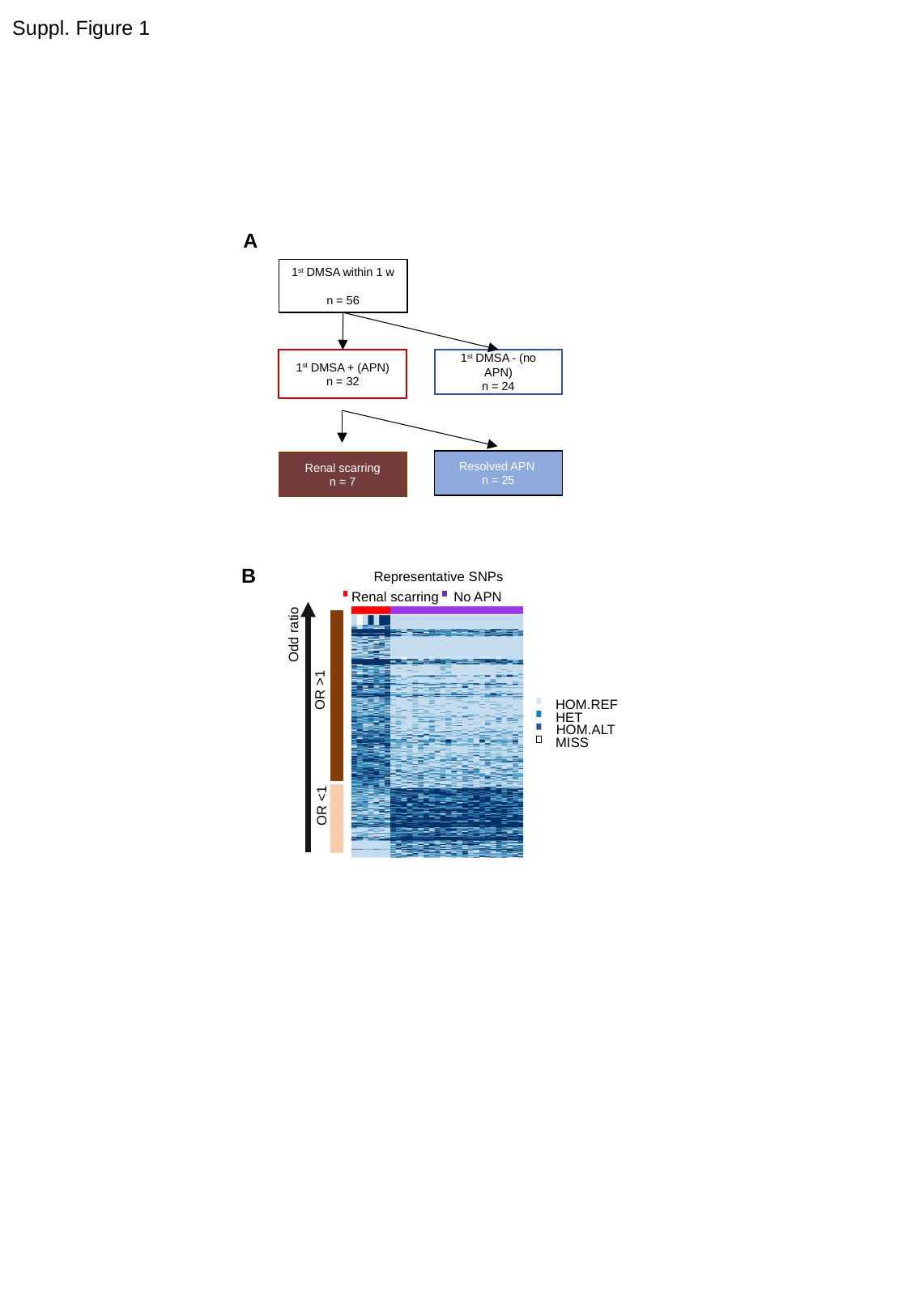

Suppl. Figure 1
A
1st DMSA within 1 w
n = 56
1st DMSA - (no APN)
n = 24
1st DMSA + (APN)
n = 32
Resolved APN
n = 25
Renal scarring
n = 7
B
Representative SNPs
Renal scarring
No APN
Odd ratio
OR >1
OR <1
HOM.REF
HET
HOM.ALT
MISS
