# Supplementary material for: Genetic determinants of renal scarring in children with febrile UTI
Source: Pediatr Nephrol. 2024 May 20;39(9):2703–15. doi: 10.1007/s00467-024-06394-6 (PMC11272715; doi:10.1007/s00467-024-06394-6)
Supplement: Supplementary file 3 — Supplementary file3 (DOCX 57 KB) [file 467_2024_6394_MOESM3_ESM.docx]

**Supplementary Table 1.** Canonical pathways of renal scarring compared to resolved APN (*p* < 0.005, adjusted *p* < 0.05)

| Ingenuity canonical pathways | Log  (p-value) | Ratio | Gene Names | P-value | Adj. p-value |
| --- | --- | --- | --- | --- | --- |
| Mitochondrial dysfunction | 5,99 | 0,0819 | APP, BACE2, COX6B2, MT-ATP6, MT-CO1, MT-CO2, MT-CO3, MT-CYB, MT-ND3, MT-ND4, MT-ND4L, MT-ND5, MT-ND6, PRKN | 1,02E-06 | 0,000446 |
| Oxidative phosphorylation | 4,81 | 0,0901 | COX6B2, MT-ATP6, MT-CO1, MT-CO2, MT-CO3, MT-CYB, MT-ND3, MT-ND4, MT-ND4L, MT-ND5 | 1,55E-05 | 0,003376 |
| Chondroitin Sulphate Biosynthesis | 3,42 | 0,103 | B3GALT6, B3GAT1, CHST1, CSGALNACT1, HS3ST2, UST | 0,00038 | 0,039377 |
| Dermatan Sulphate Biosynthesis | 3,34 | 0,1 | B3GALT6, B3GAT1, CHST1, CSGALNACT1, HS3ST2, UST | 0,000457 | 0,039377 |
| FGF signalling | 3,26 | 0,0814 | FGF12, FGF14, FGF22, FGFR2, ITPR1, MAPK1, SOS1 | 0,00055 | 0,039377 |
| Cardiac hypertrophy signalling (Enhanced) | 3,13 | 0,0369 | CACNA2D3, CACNG5, FGF12, FGF14, FGF22, FGFR2, HDAC7,I L17C,I TGA9, ITPR1, MAP3K4, MAPK1, PDE10A, PDE1C, PDE4B, PDE4C, PLCL2, PRKCE, PRKCQ, TGFBR2 | 0,000741 | 0,039377 |
| Actin Cytoskeleton signalling | 3,1 | 0,0492 | ACTN1, FGF12, FGF14, FGF22, GIT1, ITGA9, MAPK1, MYO10, SOS1, TIAM2, TRIO, VAV3 | 0,000794 | 0,039377 |
| Thymine degradation | 3,09 | 0,667 | DPYD, DPYS | 0,000813 | 0,039377 |
| Uracil Degradation II (Reductive) | 3,09 | 0,667 | DPYD, DPYS | 0,000813 | 0,039377 |
| UDP-N-acetyl-D-galactosamine Biosynthesis II | 2,95 | 0,231 | HK2, HK3, UAP1 | 0,001122 | 0,04892 |

**Supplementary Table 2**. The DNA IPA analysis of upstream regulators associated with renal scarring include involvement of mitochondrial genes (*p* < 0.005, adjusted *p* < 0.05)

| Upstream Regulator | Molecule Type | P-value | Adj. p-value | Gene name |
| --- | --- | --- | --- | --- |
| DAP3 | other | 7,44E-19 | 4,35E-16 | MT-ATP6,MT-ATP8,MT-CO1,MT-CO2,MT-CO3,MT-CYB,MT-ND3,MT-ND4,MT-ND4L,MT-ND5,MT-ND6 |
| actinonin | chemical reagent | 1,16E-14 | 3,39E-12 | MT-ATP6,MT-CO1,MT-CO2,MT-CO3,MT-CYB,MT-ND3,MT-ND4,MT-ND4L,MT-ND5,MT-ND6 |
| ALKBH7 | other | 1,36E-12 | 2,22E-10 | MT-ATP6,MT-CO1,MT-CO2,MT-CYB,MT-ND3,MT-ND4,MT-ND5,MT-ND6 |
| NSUN3 | enzyme | 1,52E-12 | 2,22E-10 | MT-ATP6,MT-CO1,MT-CO2,MT-CYB,MT-ND4,MT-ND4L,MT-ND5 |
| MT-TM | other | 1,7E-11 | 1,99E-09 | MT-CO1,MT-CO2,MT-CYB,MT-TE,MT-TK,MT-TL2,MT-TP,MT-TT |
| MT-TE | other | 8,77E-11 | 8,55E-09 | MT-ATP6,MT-CO2,MT-CYB,MT-ND4,MT-ND5,MT-ND6 |
| miR-2392 (miRNAs w/seed AGGAUGG) | mature microrna | 3,46E-10 | 2,53E-08 | HK2,MT-CO1,MT-CO2,MT-CYB,MT-ND4,MT-ND5 |
| mir-2392 | microrna | 3,46E-10 | 2,53E-08 | HK2,MT-CO1,MT-CO2,MT-CYB,MT-ND4,MT-ND5 |
| ALKBH1 | enzyme | 1,95E-09 | 1,27E-07 | MT-ATP6,MT-CO1,MT-CO2,MT-CYB,MT-ND4,MT-ND4L,MT-ND5 |
| TWNK | enzyme | 3,81E-08 | 2,12E-06 | MT-CO1,MT-CYB,MT-ND3,MT-ND4,MT-ND4L,MT-ND5,MT-ND6 |
| LONP1 | peptidase | 3,98E-08 | 2,12E-06 | MT-ATP6,MT-ATP8,MT-CO1,MT-CO2,MT-CO3,MT-CYB,MT-ND3,MT-ND4,MT-ND4L,MT-ND5,MT-ND6 |
| PAPOLA | enzyme | 5,5E-08 | 2,68E-06 | MT-ATP6,MT-CO1,MT-CO2,MT-CO3 |
| TFAM | transcription regulator | 7,56E-08 | 3,4E-06 | MT-ATP6,MT-CO1,MT-CO2,MT-CO3,MT-CYB,MT-ND3,MT-ND4,MT-ND4L,MT-ND5,MT-ND6,ZBTB16 |
| 2500002B13Rik | other | 8,92E-08 | 3,48E-06 | MT-ATP6,MT-CO1,MT-CO2,MT-CO3,MT-CYB,MT-ND4 |
| N1,N11-diethylnorspermine | chemical drug | 8,92E-08 | 3,48E-06 | MT-CO1,MT-CO2,MT-ND3,MT-ND4,MT-ND4L,MT-ND6 |
| SIRT3 | enzyme | 1,72E-07 | 6,29E-06 | MT-ATP6,MT-CO1,MT-CO2,MT-CO3,MT-CYB,MT-ND3,MT-ND4,MT-ND4L,MT-ND5,MT-ND6 |
| LRPPRC | other | 3,58E-07 | 1,23E-05 | MT-ATP6,MT-CO1,MT-ND3,MT-ND5,MT-ND6 |
| GSKJ4 | chemical reagent | 5,24E-07 | 1,7E-05 | MT-ATP6,MT-ATP8,MT-CO1,MT-CO2,MT-CO3,MT-CYB,MT-ND3,MT-ND4,MT-ND5,MT-ND6 |
| MALSU1 | other | 8,05E-07 | 2,35E-05 | MT-CO1,MT-CO2,MT-ND4,MT-ND6 |
| POLRMT | enzyme | 8,05E-07 | 2,35E-05 | MT-ATP6,MT-CO1,MT-CYB,MT-ND6 |

**Supplementary Table 3.** The DNA IPA analysis of RS compared to resolved APN. Top 10 diseases and functions associated with renal scarring.

| Categories | Diseases or functions annotation | P-value | Adj. P-value |
| --- | --- | --- | --- |
| Cancer, Organismal Injury and Abnormalities | Melanoma | 2,03E-22 | 1,02E-19 |
| Cancer, Gastrointestinal Disease, Organismal Injury and Abnormalities | Large intestine adenocarcinoma | 5,37E-22 | 1,34E-19 |
| Cardiovascular Disease, Hereditary Disorder, Metabolic Disease, Neurological Disease, Organismal Injury and Abnormalities, Psychological Disorders, Skeletal and Muscular Disorders | MELAS syndrome | 2,6E-21 | 2,98E-19 |
| Cancer, Dermatological Diseases and Conditions, Organismal Injury and Abnormalities | Cutaneous melanoma | 2,83E-21 | 2,98E-19 |
| Cancer, Gastrointestinal Disease, Organismal Injury and Abnormalities | Malignant neoplasm of large intestine | 2,98E-21 | 2,98E-19 |
| Dermatological Diseases and Conditions, Organismal Injury and Abnormalities | Skin lesion | 4,64E-21 | 3,87E-19 |
| Cancer, Organismal Injury and Abnormalities | Head and neck tumor | 6,66E-21 | 4,76E-19 |
| Cancer, Gastrointestinal Disease, Organismal Injury and Abnormalities | Gastrointestinal carcinoma | 4,35E-20 | 2,64E-18 |
| Cancer, Dermatological Diseases and Conditions, Organismal Injury and Abnormalities | Skin tumor | 4,76E-20 | 2,64E-18 |

**Supplementary Table 4.** Canonical Pathways RS compared to no APN (first negative DMSA), *p* < 0.005, adjusted *p* < 0.05

| Ingenuity Canonical Pathways | Log (p-value) | Ratio | P-value | Adj. p-value |
| --- | --- | --- | --- | --- |
| Mitochondrial Dysfunction | 6,6 | 0,0819 | 2,51E-07 | 0,000109 |
| Oxidative Phosphorylation | 5,25 | 0,0901 | 5,62E-06 | 0,00122 |
| Serotonin Receptor Signaling | 4,32 | 0,133 | 4,79E-05 | 0,006924 |
| Granzyme A Signaling | 3,94 | 0,0933 | 0,000115 | 0,010774 |
| Estrogen Receptor Signaling | 3,9 | 0,0416 | 0,000126 | 0,010774 |
| Neurovascular Coupling Signaling Pathway | 3,76 | 0,0517 | 0,000174 | 0,010774 |
| Synaptic Long-term depression | 3,76 | 0,0556 | 0,000174 | 0,010774 |
| Endocannabinoid neuronal synapse pathway | 3,44 | 0,0604 | 0,000363 | 0,019697 |
| Thymine Degradation | 3,2 | 0,667 | 0,000631 | 0,026067 |
| Uracil Degradation II (Reductive) | 3,2 | 0,667 | 0,000631 | 0,026067 |
| Netrin Signaling | 3,18 | 0,0833 | 0,000661 | 0,026067 |
| Neutrophil Extracellular Trap Signaling Pathway | 2,9 | 0,0363 | 0,001259 | 0,045531 |

**Supplementary Table 5.** Top 50 SNPs associated with dilating VUR. DNA association analysis of VUR 0-2 compared to VUR 3-5, *p* < 0.005

| Symbol | Odds ratio | P-value |
| --- | --- | --- |
| PDE4D | 5,480769231 | 1,9599E-05 |
| CNTN1 | 0,049529781 | 2,56311E-05 |
| GALNTL6 | 7,647058824 | 2,83048E-05 |
| CEP128 | 4,6 | 5,01534E-05 |
| PFKFB3 | 10,87012987 | 6,14373E-05 |
| CTD-2251F13.1 | 0,118059299 | 6,17411E-05 |
| PRKD1 | 0,118059299 | 6,17411E-05 |
| VWF | 4 | 7,07754E-05 |
| VWA7 | 23,26086957 | 9,73175E-05 |
| SAPCD1 | 23,26086957 | 9,73175E-05 |
| SAPCD1-AS1 | 23,26086957 | 9,73175E-05 |
| MSH5 | 23,26086957 | 9,73175E-05 |
| MSH5-SAPCD1 | 23,26086957 | 9,73175E-05 |
| WRAP53 | 0 | 0,000104922 |
| RP11-199F11.2 | 0 | 0,000104922 |
| TP53 | 0 | 0,000104922 |
| AGBL1-AS1 | 4,67965368 | 0,000122856 |
| AGBL1 | 4,67965368 | 0,000122856 |
| ZNF628 | 0,213691027 | 0,000122856 |
| CTD-2537I9.16 | 0,213691027 | 0,000122856 |
| NRG3 | 0,188571429 | 0,00012356 |
| CCDC178 | inf | 0,000130171 |
| RP11-83M16.6 | inf | 0,000130171 |
| RP11-434D9.2 | inf | 0,000130171 |
| RNA5SP348 | inf | 0,000130171 |
| KCNQ5 | 3,80952381 | 0,00014291 |
| MT-TT | inf | 0,000143909 |
| MT-TS2 | inf | 0,000143909 |
| MT-TS1 | inf | 0,000143909 |
| MT-TR | inf | 0,000143909 |
| MT-TK | inf | 0,000143909 |
| MT-TP | inf | 0,000143909 |
| MT-TL2 | inf | 0,000143909 |
| MT-ATP6 | inf | 0,000143909 |
| MT-ATP8 | inf | 0,000143909 |
| MT-CO1 | inf | 0,000143909 |
| MT-CO2 | inf | 0,000143909 |
| MT-CO3 | inf | 0,000143909 |
| MT-CYB | inf | 0,000143909 |
| MT-ND3 | inf | 0,000143909 |
| MT-ND4 | inf | 0,000143909 |
| MT-ND5 | inf | 0,000143909 |
| MT-ND6 | inf | 0,000143909 |
| MT-TD | inf | 0,000143909 |
| MT-TE | inf | 0,000143909 |
| MT-TH | inf | 0,000143909 |
| MT-TG | inf | 0,000143909 |
| MT-ND4L | inf | 0,000143909 |
| MITF | 4,8 | 0,000145481 |
| HUNK | 0,173453997 | 0,000156792 |

**Supplementary Table 6**. IPA canonical pathways of genes associated with VUR 3-5 (*p* < 0.005)

| Ingenuity Canonical Pathways | -log (*p*-value) | Ratio | *p*-value | Adj. *p*-value |
| --- | --- | --- | --- | --- |
| Amyotrophic Lateral Sclerosis Signaling | 4,41 | 0,103 | 3,89E-05 | 0,018402 |
| Oxidative Phosphorylation | 3,92 | 0,0991 | 0,00012 | 0,028434 |
| Neuregulin Signaling | 3,71 | 0,094 | 0,000195 | 0,030743 |
| Estrogen Receptor Signaling | 3,46 | 0,0562 | 0,000347 | 0,034412 |
| Neutrophil Extracellular Trap Signaling Pathway | 3,41 | 0,0557 | 0,000389 | 0,034412 |
| Mitochondrial Dysfunction | 3,36 | 0,076 | 0,000437 | 0,034412 |
| Granzyme A Signaling | 3,21 | 0,107 | 0,000617 | 0,041664 |

**Supplementary Table 7.** IPA presenting upstream regulators associated with infants with dilating VUR (i.e. VUR 0-2 compared to VUR 3-5) (*p<* 0.005, adj. *p* <0.05)

| Upstream regulator | Molecule type | P-value | Adj. p-value | Gene names |
| --- | --- | --- | --- | --- |
|  |  |  |  | MT-CO1, MT-CO2, MT-CYB, MT-ND1, MT-RNR1, MT-RNR2, MT-TE, MT |
| MT-TM | other | 3,25E-25 | 2,07E-22 | TK, MT-TL1, MT-TL2, MT-TM, MT-TP, MT-TQ, MT-TT, MT-TY |
|  |  |  |  | MT-ATP6, MT-ATP8, MT-CO1, MT-CO2, MT-CO3, MT-CYB , MT |
| DAP3 | other | 6,12E-22 | 1,95E-19 | ND1, MT-ND2,MT-ND3,MT-ND4,MT-ND4L,MT-ND5,MT-ND6 |
|  | chemical |  |  | MT-ATP6,MT-CO1,MT-CO2,MT-CO3,MT-CYB,MT-ND1,MT- |
| actinonin | reagent | 1,47E-16 | 3,13E-14 | ND2,MT-ND3,MT-ND4,MT-ND4L,MT-ND5,MT-ND6 |
|  |  |  |  | MT-ATP6,MT-CO1,MT-CO2,MT-CYB,MT- |
|  |  |  |  | ND1,MT-ND2,MT-ND3,MT-ND4,MT-ND5,MT- |
| ALKBH7 | other | 3,12E-15 | 4,98E-13 | ND6 |
|  |  |  |  | MT-ATP6,MT-CO1,MT-CO2,MT-CYB,MT- |
| NSUN3 | enzyme | 9,13E-14 | 1,16E-11 | ND2,MT-ND4,MT-ND4L,MT-ND5 |
|  |  |  |  | MT-ATP6,MT-CO2,MT-CYB,MT- |
| MT-TE | other | 3,92E-12 | 4,17E-10 | ND1,MT-ND4,MT-ND5,MT-ND6 |
|  |  |  |  | MT-CO1,MT-CO2,MT-ND1,MT- |
|  |  |  |  | ND2,MT-ND6,MT-RNR1,MT- |
| MRPL12 | other | 3,07E-11 | 2,8E-09 | RNR2 |
|  |  |  |  | MT-ATP6,MT-CO1,MT-CO2,MT-CO3,MT-CYB,MT-ND1,MT-ND2,MT- |
| SIRT3 | enzyme | 1,34E-10 | 1,07E-08 | ND3,MT-ND4,MT-ND4L,MT-ND5,MT-ND6,PPARGC1A,TFRC,TP53 |
|  |  |  |  | MT-ATP6,MT-CO1,MT-CO2,MT-CYB,MT- |
| ALKBH1 | enzyme | 9,94E-10 | 6,86E-08 | ND2,MT-ND4,MT-ND4L,MT-ND5 |
|  |  |  |  | MT-ATP6,MT-CO1,MT-ND1,MT- |
| LRPPRC | other | 1,19E-09 | 6,86E-08 | ND2,MT-ND3,MT-ND5,MT-ND6 |

IPA presenting upstream regulators associated to infants with dilating VUR (VUR 0-2 compared to VUR 3-5) and mitochondrial genes. P<0.005, adj P<0.05.

**Supplementary Table 8.** A gene list of polymorphic genes associated with recurrent UTI (*p* < 0.005).

|  |  |  |  | Recurrent vs Non-recurrent | | Recurrent vs SweGen | |
| --- | --- | --- | --- | --- | --- | --- | --- |
| Gene symbol | Coordinate (Hg19) | Ref. / Alt. allele | SweGen AF | OR | P-val. | OR | P-val. |
| CTB-57H20.1 | 5:143058402 | C/T | 0,2115 | 5,24 | 4,53531E-06 | 2,11 | 0.00508 |
| MIR5197 | 5:143058402 | C/T | 0,2115 | 5,24 | 4,53531E-06 | 2,11 | 0.00508 |
| NR5A2 | 1:200007432 | A/G | 0,2455 | 0,16 | 4,94482E-06 | 0,28 | 0.00103 |
| HCG22 | 6:31019970 | A/G | 0.147 | 5,00 | 1,48288E-05 | 2,90 | 0.00009 |
| AC073283.4 | 2:47423575 | A G | 0.784 | 5,77 | 1,69595E-05 | 3,03 | 0.00488 |
| CTC-756D1.2 | 8:23330539 | C/T | 0,1315 | 5,76 | 4,04853E-05 | 2,37 | 0.00425 |
| PELI2 | 14:56725866 | G/A | 0,1735 | 4,29 | 4,16313E-05 | 2,53 | 0.00047 |
| RP1-167F1.2 | 6:19560246 | T/G | 0.859 | 17,89 | 5,18548E-05 | 11,65 | 0.00063 |
| RP11-539G18.2 | 4:39637826 | C/T | 0,5020 | 0,30 | 5,68461E-05 | 0,50 | 0.00555 |
| SMIM14 | 4:39637826 | C/T | 0,5020 | 0,30 | 5,68461E-05 | 0,50 | 0.00555 |
| PDE4D | 5:59592945 | C/T | 0,6110 | 0,30 | 7,88911E-05 | 0,57 | 0.01975 |
| WIPF3 | 7:29889233 | G/A | 0,5965 | 3,50 | 7,95336E-05 | 2,37 | 0.00199 |
| AC131012.1 | 12:76117991 | T/G | 0,0445 | inf | 8,03754E-05 | 2,68 | 0.01728 |
| RP11-114H23.1 | 12:76117991 | T/G | 0,0445 | inf | 8,03754E-05 | 2,68 | 0.01728 |
| RP11-298E9.5 | 10:3264809 | C/T | 0.467 | 3,20 | 9,82353E-05 | 2,14 | 0.00246 |
| AC012593.1 | 2:35299844 | C/T | 0,2285 | 3,39 | 0,00011 | 2,70 | 0.00009 |
| AC018685.1 | 2:35299844 | C/T | 0,1515 | 7,19 | 0,00012 | 1,35 | 0.31765 |
| LINC00243 | 6:30768412 | C/T | 0,3540 | 3,23 | 0,00012 | 1,93 | 0.00755 |
| EIF3E | 8:109280487 | A/G | 0,2095 | 3,36 | 0,00012 | 3,19 | 3.65516E-06 |
| PRKCE | 2:46205320 | C/T | 0,5355 | 0,31 | 0,00013 | 0,62 | 0.05419 |
| CTC-340D7.1 | 5:68220909 | A/G | 0,1960 | 4,40 | 0,00013 | 1,80 | 0.03420 |
| TSSC2 | 11:3401374 | T/C | 0,8415 | 0,23 | 0,00013 | 0,43 | 0.00294 |
| ZNF195 | 11:3401374 | T/C | 0,8415 | 0,23 | 0,00013 | 0,43 | 0.00294 |
| KALRN | 3:124121911 | A/G | 0,2055 | 3,62 | 0,00013 | 2,46 | 0.00057 |
| RNU6-143P | 3:124121911 | A/G | 0,2055 | 3,62 | 0,00013 | 2,46 | 0.00057 |

**Supplementary Table 9**. IPA canonical pathways analysis of genes associated with infants with UTI recurrences

| Ingenuity Canonical Pathways | -log(*p*-value) | Ratio | *p*-value | Adj. *p*-value |
| --- | --- | --- | --- | --- |
| Dilated Cardiomyopathy Signaling Pathway | 7,36 | 0,113 | 4,36516E-08 | 2,13893E-05 |
| Calcium Signaling | 6,93 | 0,0909 | 1,1749E-07 | 2,8785E-05 |
| Nitric Oxide Signaling in the Cardiovascular System | 6,33 | 0,117 | 4,67735E-07 | 7,09947E-05 |
| Synaptogenesis Signaling Pathway | 6,15 | 0,073 | 7,07946E-07 | 7,09947E-05 |
| Gustation Pathway | 6,14 | 0,0887 | 7,24436E-07 | 7,09947E-05 |
| nNOS Signaling in Skeletal Muscle Cells | 6,02 | 0,188 | 9,54993E-07 | 7,79911E-05 |
| Cellular Effects of Sildenafil (Viagra) | 5,87 | 0,1 | 1,34896E-06 | 9,44274E-05 |
| Amyotrophic Lateral Sclerosis Signaling | 5,72 | 0,112 | 1,90546E-06 | 0,000116709 |
| Netrin Signaling | 5,37 | 0,139 | 4,2658E-06 | 0,000232249 |
| Oxytocin Signaling Pathway | 5,24 | 0,0709 | 5,7544E-06 | 0,000281966 |
| Circadian Rhythm Signaling | 5 | 0,0709 | 0,00001 | 0,000445455 |
| White Adipose Tissue Browning Pathway | 4,88 | 0,0942 | 1,31826E-05 | 0,000538288 |
| Opioid Signaling Pathway | 4,73 | 0,0679 | 1,86209E-05 | 0,000666911 |
| Neurovascular Coupling Signaling Pathway | 4,72 | 0,0733 | 1,90546E-05 | 0,000666911 |
| Mitochondrial Dysfunction | 4,52 | 0,0819 | 3,01995E-05 | 0,000986518 |
| Oxidative Phosphorylation | 4,42 | 0,0991 | 3,80189E-05 | 0,00116433 |
| Insulin Secretion Signaling Pathway | 4,37 | 0,0662 | 4,2658E-05 | 0,001229553 |
| Protein Kinase A Signaling | 4,26 | 0,056 | 5,49541E-05 | 0,001495972 |
| Dopamine-DARPP32 Feedback in cAMP Signaling | 4,12 | 0,0753 | 7,58578E-05 | 0,001956332 |
| GNRH Signaling | 3,99 | 0,0733 | 0,000102329 | 0,002507068 |
| Estrogen Receptor Signaling | 3,85 | 0,0538 | 0,000141254 | 0,003235824 |
| Synaptic Long Term Depression | 3,83 | 0,0707 | 0,000147911 | 0,003235824 |
| Corticotropin Releasing Hormone Signaling | 3,81 | 0,0789 | 0,000154882 | 0,003235824 |
| Role of NFAT in Cardiac Hypertrophy | 3,8 | 0,067 | 0,000158489 | 0,003235824 |
| Cardiac β-adrenergic Signaling | 3,69 | 0,0722 | 0,000204174 | 0,004001806 |

**Supplementary Table 10.** IPA presenting Tox functions associated with recurrent UTI. *p* < 0.005. Liver, heart and kidneys are affected.

| Categories | Diseases or Functions Annotation | *p* - value | Adj. *p*-value | # Molecules |
| --- | --- | --- | --- | --- |
| Liver Hyperplasia/Hyperproliferation | Liver carcinoma | 7,52E-22 | 2,2635E-19 | 262 |
| Liver Hyperplasia/Hyperproliferation | Liver cancer | 3,1E-21 | 4,6655E-19 | 267 |
| Liver Hyperplasia/Hyperproliferation | Liver tumor | 3,04E-19 | 3,0501E-17 | 288 |
| Cardiac Arteriopathy | Disorder of coronary artery | 1,07E-10 | 8,0518E-09 | 35 |
| Cardiac Arteriopathy | Coronary artery disease | 5,1E-09 | 3,0702E-07 | 26 |
| Cardiac Arrythmia, Tachycardia | Tachycardia | 0,0000834 | 0,0041839 | 11 |
| Heart Failure | Acute heart failure | 0,000176 | 0,00695644 | 8 |
| Cardiac Arrythmia | Arrhythmia | 0,000187 | 0,00695644 | 21 |
| Cardiac Arrythmia | Long-QT syndrome | 0,000208 | 0,00695644 | 7 |
| Nephrosis | Nephrosis | 0,000273 | 0,0082173 | 14 |
| Cardiac Arrythmia | Arrhythmia of heart ventricle | 0,000331 | 0,00905736 | 10 |
| Cardiac Arrythmia | Familial arrhythmogenic right ventricular dysplasia type 2 | 0,000493 | 0,01141485 | 2 |
| Cardiac Arrythmia | Short-QT syndrome 4 | 0,000493 | 0,01141485 | 2 |
| Cardiac Arrythmia | Familial ventricular arrhythmia | 0,000561 | 0,01157847 | 5 |
| Heart Failure | Sudden cardiac death | 0,000577 | 0,01157847 | 4 |
| Cardiac Arrythmia | Arrhythmogenic right ventricular cardiomyopathy | 0,000652 | 0,01226575 | 6 |
| Cardiac Arrythmia, Tachycardia | Catecholaminergic polymorphic ventricular tachycardia | 0,000882 | 0,01488278 | 4 |
| Cardiac Arrythmia, Tachycardia | Ventricular tachycardia | 0,00089 | 0,01488278 | 8 |
| Cardiac Arrythmia | Short QT syndrome | 0,00117 | 0,01853526 | 3 |
| Pulmonary Hypertension | Pulmonary Hypertension | 0,00181 | 0,02637333 | 12 |
| Cardiac Dilation, Cardiac Enlargement | Primary dilated cardiomyopathy | 0,00184 | 0,02637333 | 9 |
| Cardiac Arrythmia, Congenital Heart Anomaly | Long QT syndrome 1 | 0,00207 | 0,02832136 | 3 |
| Cardiac Arrythmia | Syndromic arrhythmia | 0,00217 | 0,0283987 | 9 |
| Cardiac Enlargement | Enlargement of heart chamber | 0,00241 | 0,03022542 | 14 |
| Cardiac Arrythmia | Wolff-Parkinson-White syndrome | 0,00252 | 0,0303408 | 6 |

**Supplementary Table 11.** IPA presenting canonical pathways of common genes of VUR 3-5, rec UTI and RS

| Canonical Pathways - common RS, VUR 3-5, rec UTI | |
| --- | --- |
| Ingenuity Canonical Pathways | -log(p-value) |
| Cardiac Hypertrophy Signaling (Enhanced) | 2,54E-01 |
| Axonal Guidance Signaling | 2,72E-01 |
| Pulmonary Fibrosis Idiopathic Signaling Pathway | 4,14E-01 |
| Synaptogenesis Signaling Pathway | 4,26E-01 |
| CLEAR Signaling Pathway | 4,60E-01 |
| Protein Ubiquitination Pathway | 4,75E-01 |
| Signaling by Rho Family GTPases | 4,83E-01 |
| Actin Cytoskeleton Signaling | 5,16E-01 |
| RHOGDI Signaling | 5,53E-01 |
| Clathrin-mediated Endocytosis Signaling | 5,74E-01 |
| Regulation of the Epithelial-Mesenchymal Transition Pathway | 5,98E-01 |
| Regulation Of the Epithelial Mesenchymal Transition By Growth Factors Pathway | 6,04E-01 |
| Macrophage Classical Activation Signaling Pathway | 6,10E-01 |
| Tumor Microenvironment Pathway | 6,31E-01 |
| GŒ±12/13 Signaling | 7,46E-01 |
| Airway Pathology in Chronic Obstructive Pulmonary Disease | 7,93E-01 |
| Bladder Cancer Signaling | 8,00E-01 |
| FGF Signaling | 9,21E-01 |
| BAG2 Signaling Pathway | 9,30E-01 |
| Serotonin Degradation | 9,99E-01 |
| Superpathway of Melatonin Degradation | 1,02E+00 |
| Nicotine Degradation II | 1,03E+00 |
| Melatonin Degradation I | 1,06E+00 |
| Dermatan Sulfate Biosynthesis | 1,07E+00 |
| Chondroitin Sulfate Biosynthesis | 1,08E+00 |
| Nicotine Degradation III | 1,08E+00 |
| Chondroitin Sulfate Biosynthesis (Late Stages) | 1,15E+00 |
| Thyroid Hormone Metabolism II (via Conjugation and/or Degradation) | 1,24E+00 |
| Purine Nucleotides Degradation II (Aerobic) | 1,56E+00 |
| Adenosine Nucleotides Degradation II | 1,63E+00 |
| Parkinson's Signaling | 1,63E+00 |
| Purine Ribonucleosides Degradation to Ribose-1-phosphate | 1,88E+00 |
| Adenine and Adenosine Salvage III | 1,99E+00 |
| Chondroitin and Dermatan Biosynthesis | 2,05E+00 |
| HER-2 Signaling in Breast Cancer | 2,34E+00 |
| Glucocorticoid Receptor Signaling | 4,70E+00 |
| Neutrophil Extracellular Trap Signaling Pathway | 5,68E+00 |
| Estrogen Receptor Signaling | 5,71E+00 |
| Sirtuin Signaling Pathway | 6,68E+00 |
| Granzyme A Signaling | 7,07E+00 |
| Mitochondrial Dysfunction | 1,18E+01 |
| Oxidative Phosphorylation | 1,33E+01 |

**Supplementary Table 12.** Venn analysis, common genes RS, dilating VUR and recurrent UTI showing Tox function common genes RS, VUR 3-5, rec UTI

| Categories | Diseases or Functions Annotation | p-value |
| --- | --- | --- |
| Congenital Heart Anomaly | Tetralogy of Fallot | 2,85E-06 |
| Cardiac Dysfunction, Congenital Heart Anomaly | Bicuspid aortic valve | 2,22E-04 |
| Congenital Heart Anomaly | Congenital heart disease | 3,85E-04 |
| Cardiac Dilation, Cardiac Enlargement | Idiopathic mitochondrial dilated cardiomyopathy | 1,48E-03 |
| Cardiac Arteriopathy | Coronary artery disease | 1,01E-02 |
| Cardiac Enlargement | Right ventricular hypertrophy | 5,49E-02 |
| Liver Inflammation/Hepatitis | Chronic hepatitis C | 1,19E-01 |
| Pulmonary Hypertension | Pulmonary hypertensive arterial disease | 1,25E-01 |
| Glomerular Injury, Renal Fibrosis | Fibrosis of kidney | 2,20E-01 |
| Liver Damage | Hepatic injury | 2,28E-01 |
| Hepatocellular carcinoma, Liver Hyperplasia/Hyperproliferation | Hepatocellular carcinoma | 2,83E-01 |
| Cardiac Necrosis/Cell Death | Cell death of cardiomyocytes | 3,63E-01 |
| Cardiac Enlargement | Enlargement of heart | 4,08E-01 |
| Liver Hyperplasia/Hyperproliferation | Liver carcinoma | 5,02E-01 |

IPA annotations of Tox functions associated with the common genes of the clinical risk groups of infants with renal scarring, dilating VUR and recurrent UTIs.
